# Meta-analysis of the association between interleukin-17 and ischemic cardiovascular disease

**DOI:** 10.1186/s12872-024-03897-w

**Published:** 2024-05-16

**Authors:** Yu Miao, Tao Yan, Jia Liu, Chunfa Zhang, Jinli Yan, Lei Xu, Nan Zhang, Xingguang Zhang

**Affiliations:** Department of Health Statistics, Public Health College, Inner Mongolia Medical University, Hohhot, Inner Mongolia Province, 010000 China

**Keywords:** Interleukin 17, Ischemic cardiovascular disease, Meta-analysis

## Abstract

**Background:**

Interleukin-17 (IL-17) has been hypothesized to be involved in ischemic cardiovascular disease (ICVD). However, the association of IL-17 with ICVD remained unclear. The aim of this study was to systematically analyze the available evidence regarding the association between IL-17 and ICVD.

**Methods:**

We searched the PubMed, Web of Science, Cochrane Library, and Embase databases up to October 2023 to identify publications on the association between IL-17 and ICVD. The merged results were analyzed using a random effects model for meta-analysis and subgroup analysis.

**Results:**

A total of 955 publications were initially identified in our search and screened; six studies were eventually included in the analysis. The average age of study participants was 60.3 ± 12.6 years and 65.5% were men. There was a high degree of heterogeneity among studies. The results showed that IL-17 level were higher in the case group than those in the control group (standardized mean difference, SMD = 1.60, 95% confidence interval (95% CI): 0.53–2.66, *P* = 0.003). In sensitivity analysis, the merged results showed good robustness. Additionally, subgroup analysis showed that race and ethnicity, sample size, and detection methods were significant factors influencing heterogeneity in the published studies.

**Conclusion:**

Our finding revealed that increased IL-17 level contributed to the development of ICVD, suggesting IL-17 as a potential risk marker. Further research is needed to establish IL-17 as a therapeutic biomarker of ICVD.

**Supplementary Information:**

The online version contains supplementary material available at 10.1186/s12872-024-03897-w.

## Background

Atherosclerotic cardiovascular disease is mainly mediated by infiltrated inflammatory cells. Therefore, inflammation plays a key role in the formation, development, and complications of atherosclerosis [1]. As inflammatory markers, various cytokines are involved in the formation of atherosclerosis [2, 3]. Studies have shown that IL-17 has a major role in various inflammatory diseases via participating in the regulation of chronic inflammation, thrombosis formation, and atherosclerosis. IL-17 can stimulate various cells to release adhesion molecules and pro-inflammatory cytokines, among which IL-17A is important in the inflammatory response [4].

At present, the role of IL-17 in coronary heart disease remains controversial. Most studies support that the elevated level of IL-17 is considered to play a crucial role in ICVD. According to reports, IL-17 level are increased in patients with acute coronary syndrome (ACS), which poses cardiovascular disease’ risks [5]. This may be due to the interaction between endothelial cells and IL-17 at the lesion prone site, which triggers local inflammatory response at the early stage of atherosclerosis, leads to plaque instability and ACS. However, it has also been suggested that IL-17 may have a protective role in atherosclerosis [6]. In a prospective study, serum IL-17 level was associated with mortality and recurrence in patients with acute myocardial infarction (AMI), lower IL-17 level was a risk factor for death or recurrent AMI [7]. A clinical trial found that plasma IL-17A level was significantly higher in patients with coronary heart disease (CHD) than in healthy controls and were positively correlated with platelet aggregation level. In patients with ACS, IL-17 level in those with AMI showed a significant upward trend compared with the group that had angina. The research results also indicated that concentrations of IL-17 in the plasma of patients with unstable plaques were significantly higher than those in patients with stable plaques. Additionally, IL-17 level have been found to be higher in patients with severe complex lesions [8]. Therefore, it can be inferred that in patients with coronary heart disease, high IL-17 expression may be an important factor led to plaque instability or even rupture [4]. Most studies supported that IL-17 level are positively correlated with the severity of vascular lesions, which can more sensitively reflect the acute degree and severity of CHD [9]. However, a small number of studies pointed to the exact opposite conclusion [7, 10, 11]. In view of the controversial relationship between IL-17 and ICVD and the lack of relevant studies, we conducted a meta-analysis to explore the correlation between IL-17 and ICVD. We aimed to provide more detailed reference information to improve the monitoring of IL-17 level in patients with ICVD in the clinical setting.

## Methods

### Search strategy

We conducted a search using the PubMed, Web of Science, Cochrane Library, and Embase databases to identify publications in English on the relationship between IL-17 and ICVD up to October 2023. We have screened the references of the included studies and not used any filters. The search strategy which we used included the following subject headings and search formula: ("Interleukin 17" OR "IL-17" OR "interleukin 17″) AND ("ischemi*" AND ("Cardiovascular Diseases" OR "cardiovascular disease*" OR "myocardial infarction" OR "stable angina" OR "unstable angina" OR "arrhythmias, cardiac" OR "cardiac arrhythmias" OR "arrhythmia*")). The prosepro registration ID for this review is CRD42023472849.

### Literature screening and data extraction

All the retrieved literature was imported into EndnoteX9 software and independently screened by two researchers. The inclusion criteria were as follows: (1) case–control or prospective studies on IL-17 level and ICVD; (2) research focused on the relationship between IL-17 level and ICVD; (3) publications in English; (4) participants' IL-17 level were reported, or relevant statistics or effect values that reflect the relationship between IL-17 and ICVD were provided. The exclusion criteria were: (1) non-population research; (2) conference abstracts or review articles; (3) duplicated publications or literature with duplicated data; (4) articles with unclear data or those for which we were unable to obtain the original text. The retrieved articles were screened according to the inclusion and exclusion criteria. After the initial screened, the original full text was reviewed and irrelevant publications were eliminated. Then, we performed data extraction and established a database using Excel (Microsoft Corporation, Redmond, WA, USA). We used standardized data tables to extract the following information: first author, publication year, number of cases and controls, study design, participants’ race and ethnicity, participant demographics, measurement methods, and participants’ IL-17 level.

### Literature quality evaluation

We followed the quality evaluation method recommended by the Cochrane Collaboration, namely, the Newcastle–Ottawa Scale [12, 13], to assess the quality and risk of bias in the included studies, for which the relevant data were extracted. We followed PRISMA 2020 guidelines.

### Statistical analysis

The data from included studies were analyzed using Cochrane’s Review Manager (RevMan) version 5.2 and Stata 12.0 software (StataCorp LLC, College Station, TX, USA). SMD was used for the measurement data as the combined effect size, and point estimates and 95% CIs were given. The *I*^*2*^ test was used to evaluate the statistical heterogeneity of the included studies. *P* < 0.05 and *I*^*2*^ ≥ 50% indicated high heterogeneity among studies, and a random effects model was used to combine the effect sizes. Sensitivity analysis was carried out using the leave-one-out method to evaluate the robustness and reliability of the combined results of meta-analysis. Used Begg's rank correlation and Egger's linear regression methods to test publication bias. A funnel plot was used to determine whether publication bias existed. We conducted subgroup analysis stratified by race and ethnicity, sample size, and protein measurement method.

## Results

### Basic information of the included studies

**Fig. 1:**
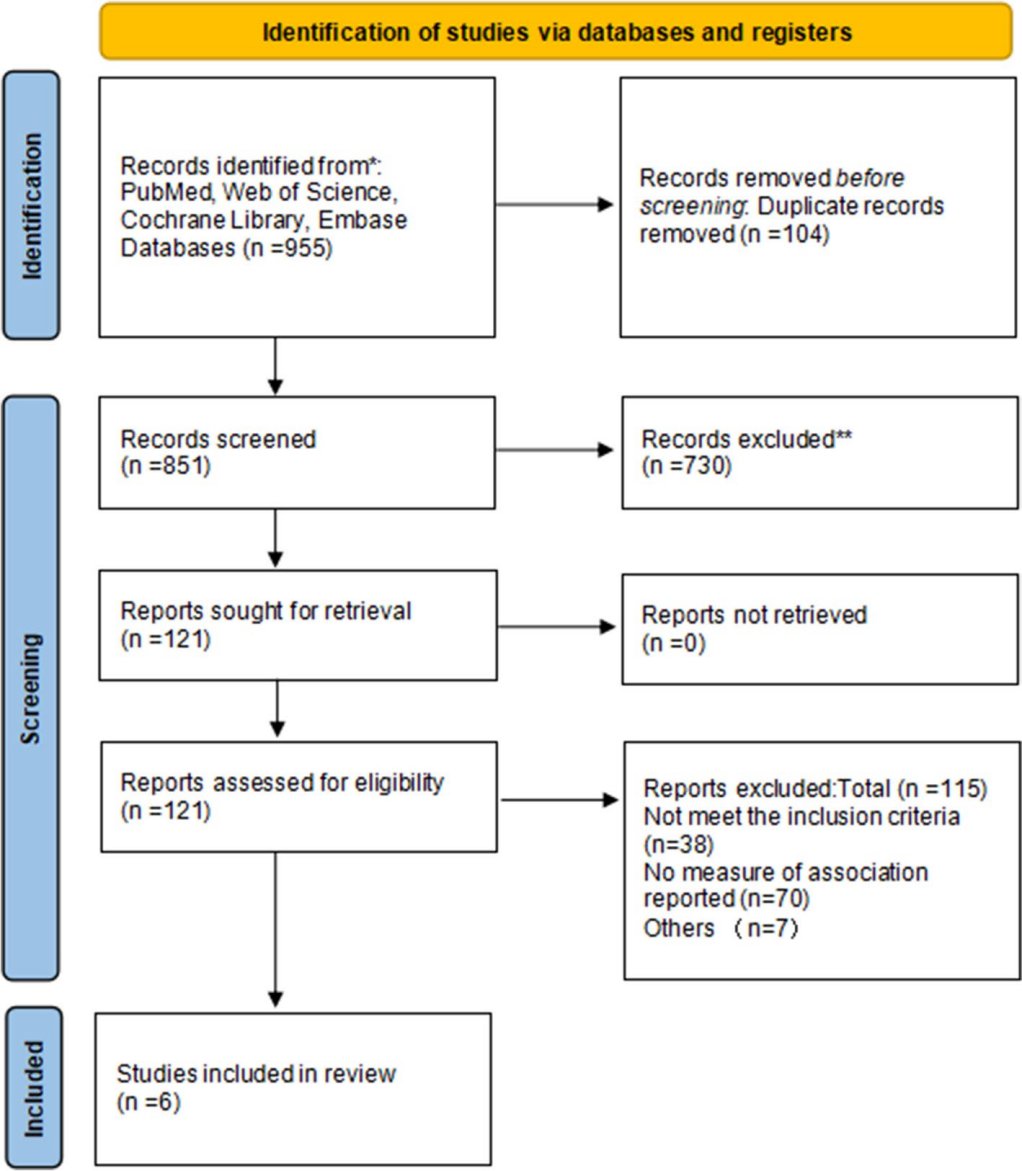
Literature screening process

A total of 955 relevant publications were retrieved, and 121 were retained in preliminary screened of article titles and abstracts. After a full-text review according to our criteria, six case–control studies were finally included in the present meta-analysis. The specific process of literature screening was shown in Fig. 1.

A total of 956 participants were included among all studies, with 701 cases and 255 healthy controls. Studies reported IL-17 concentrations in the case and control groups were all of moderate to high quality. The study characteristics and IL-17 level at baseline in each study were shown in Table 1. After comprehensive evaluation of each study, we found that Newcastle–Ottawa Scale scores in the six studies reached 5 points or above, indicating that the overall quality of the included articles was moderate to high [13], as shown in Table 2.

**Table 1:** Characteristics of the included studies

Author, Year	Design	Area	Disease	Sample		Sex(M/F)		Age(Year)		IL-17 concentration			Methods
				**Case**	**Control**	**Case**	**Control**	**Case**	**Control**	**Case**	**Control**	**Unit**	
Sheng Liu 2022 [14]	CCS	China	CHD	393	109	293/100	55/54	60.2 ± 10.3	59.1 ± 9.6	4.19 (1.97–9.06)	4.22 (2.50–7.81)	pg/ml	FCM
Hanmei Lu 2022 [15]	CCS	China	CHD	150	50	113/37	38/12	61.7 ± 9.4	60.1 ± 4.0	58.6(42.3–85.5)	43.6 (33.4–53.9)	pg/ml	ELISA
Mateusz G 2014 [16]	CCS	United States	CI	43	26	18/25	10/16	70.0 (60.0, 73.0)	55.5 (50.2, 61.0)	7.01 ± 2.30	4.78 ± 3.10	pg/ml	FCM
Maria del 2013 [17]	CCS	German	AMI	20	20	12/8	12/8	68 (47, 92)	63 (43, 86)	271.9 ± 158	22.75 ± 7.16	pg/ml	FCM
Qing Li 2013 [18]	CCS	China	CI	37	30	22/15	18/12	61.9 ± 15.8	57.6 ± 16.5	54.87 ± 12.13	16.40 ± 3.81	pg/ml	ELISA
Satwat Hashmi 2006 [19]	CCS	China	CHD	58	20	44/14	14/6	56.2 ± 11.6	51.7 ± 7.2	340.3 ± 162.75	257.16 ± 71.37	pg/ml	ELISA

Disaggregated data are expressed as number (n) and continuous data are expressed as mean ± standard deviation or median (interquartile range)

*IL-17* interleukin 17, *ELISA* enzyme-linked immunosorbent assay, *FCM* flow cytometry, *CCS* case–control study, *CHD* coronary heart disease, *AMI* acute myocardial infarction, *CI* cerebral infarction

**Table 2:** Quality evaluation for the included studies

Study	Research object selection				Comparability between groups	Exposure or outcome evaluation			total points
	**(1)**	**(2)**	**(3)**	**(4)**	**(5)**	**(6)**	**(7)**	**(8)**	
Sheng Liu,2022 [14]	1	1	0	1	2	1	1	0	7
Hanmei Lu,2022 [15]	1	1	1	1	1	1	1	1	8
Mateusz G.Adamski,2014 [16]	1	1	1	1	1	1	1	0	7
Maria del,2013 [17]	1	0	1	1	2	0	0	0	5
Qing Li,2013 [18]	1	1	1	1	2	1	1	0	8
Satwat Hashmi,2006 [19]	1	1	1	1	2	0	0	0	6

Full score is 9; items with a score of 1 are as follows. (1) Determine whether the case is appropriate. (2) Representativeness of cases. (3) Choice of controls. (4) Determination of the comparison group. (6) Determination of exposure factors. (7) The same method used to determine exposure factors of cases and controls. (8) No response rate. The item with a score of 2 is (5) Comparability of cases and controls was considered in design and statistical analysis

### Meta-analysis of quantitative variables

#### Combined effect size results

**Fig. 2:**
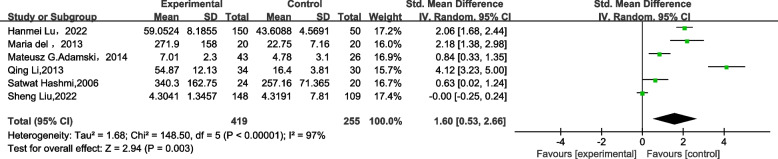
Forest map of the difference in IL-17 level between patients with ICVD and healthy controls. IL-17, interleukin 17; ICVD, ischemic cardiovascular disease; SD, standard deviation; CI, confidence interval; IV, inverse variance

Among the six included studies, the median and interquartile range were converted to mean ± standard deviation. We used RevMan 5.2 to calculate the SMD as an index of the merger effect. The test of heterogeneity indicated high heterogeneity among studies (*I*^*2*^ = 97%*, P* < 0.001). The IL-17 level in the case group was higher than those in the control group, and the difference between groups was statistically significant (SMD = 1.60, 95% CI: 0.53–2.66, *P* = 0.003*,* Fig. 2).

#### Sensitivity analysis

**Fig. 3:**
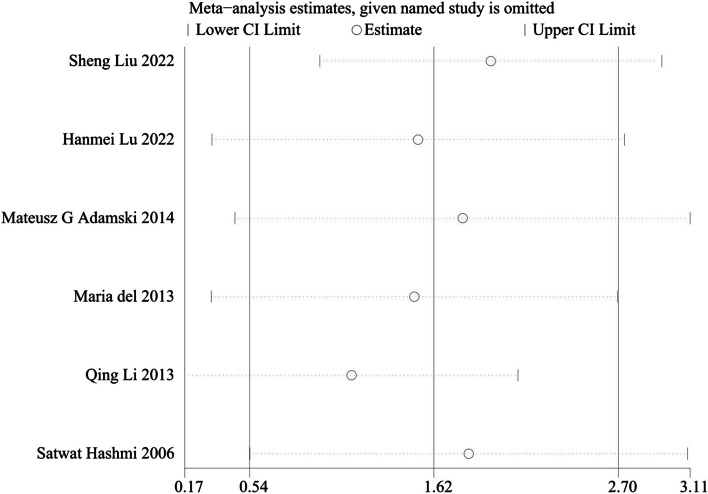
Sensitivity analysis of the difference in IL-17 level between patients with ICVD and healthy controls. IL-17, interleukin 17; ICVD, ischemic cardiovascular disease; CI, confidence interval

**Fig. 4:**
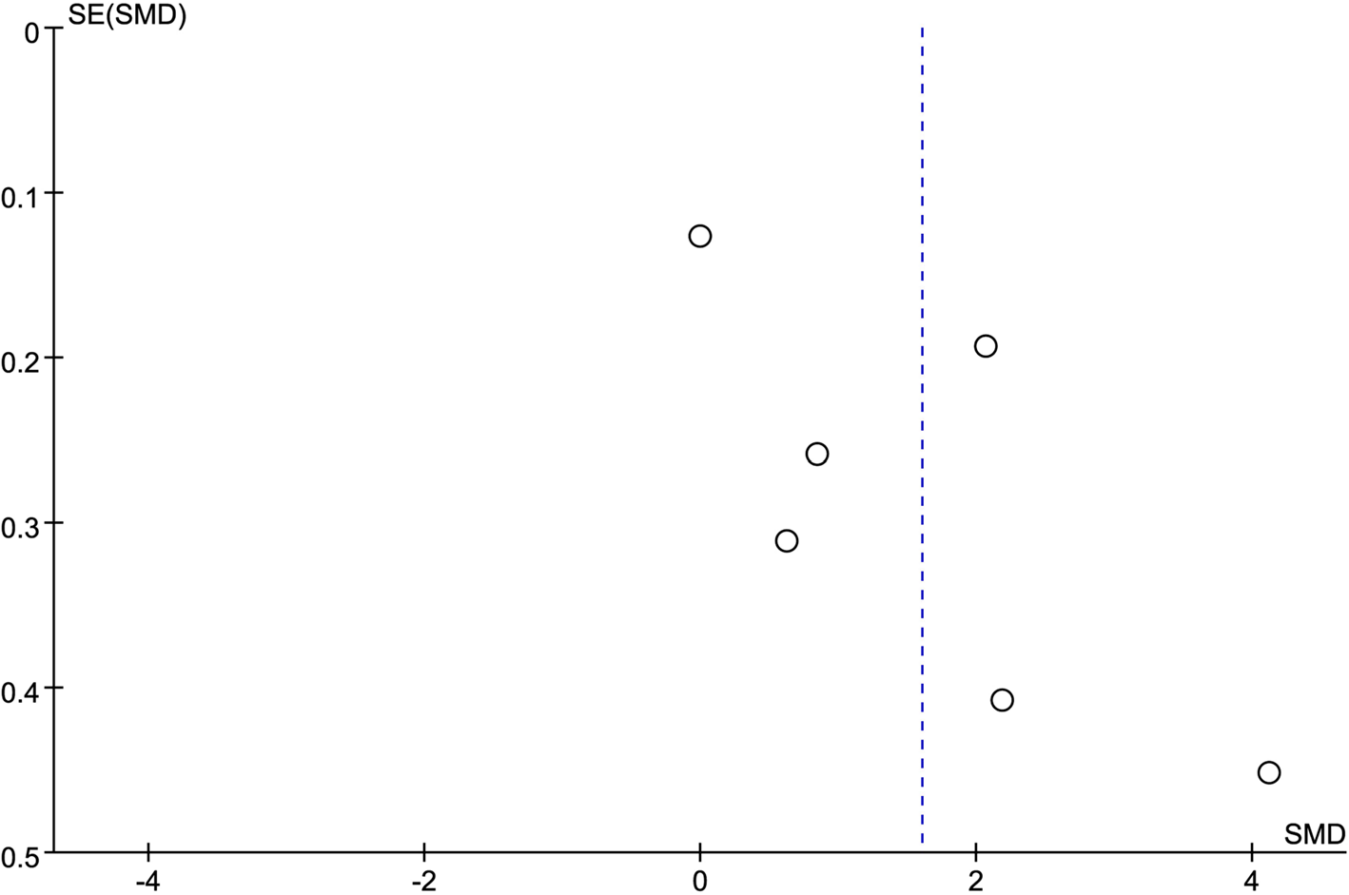
Funnel plot of publication bias for differences in IL-17 level between ICVD patients and controls. IL-17, interleukin 17; ICVD, ischemic cardiovascular disease; SE, standard error; SMD, standardized mean difference

The results of sensitivity analysis showed that the six studies were all within the 95% CI, indicating good stability of the SMD in the combined effect results. Asymmetry tests of publication bias showed *P* = 0.260 using Begg’s rank correlation method and *P* = 0.102 using Egger’s linear regression method, further confirming that there was no obvious publication bias (Fig. 3). The sensitivity analysis confirmed the robustness of the merged results. The funnel plot was symmetric and indicated there was no evidence of publication bias (Fig. 4).

**Fig. 5:**
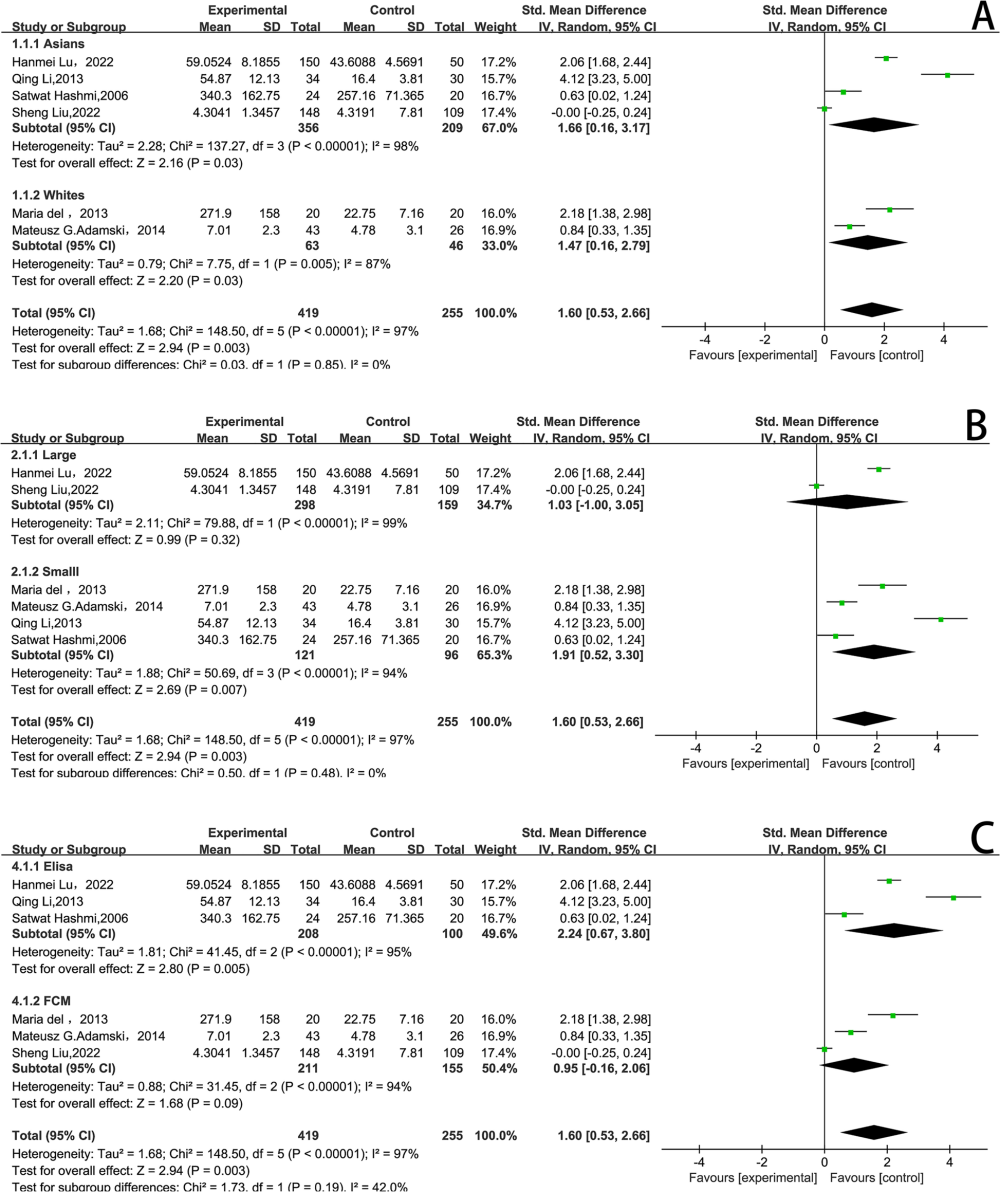
Subgroup analyses of differences in IL-17 level between ICVD patients and controls. **A** race; **B** sample size; **C** detection method. ELISA, enzyme-linked immunosorbent assay; FCM, flow cytometry; df, degrees of freedom; SD, standard deviation; CI, confidence interval; IV, inverse variance

In the subgroup analysis stratified by race and ethnicity, we found that IL-17 level were higher in the case group than in controls among Asians (SMD = 1.66, 95% CI: 0.16–3.17, *P* = 0.03) and Whites (SMD = 1.47, 95% CI: 0.16–2.79, *P* = 0.03). Additionally, high IL-17 level were associated with susceptibility to ICVD in the small-sample subgroup (SMD = 1.91, 95% CI: 0.52–3.30, *P* = 0.007) but not in the large-sample subgroup. Furthermore, in subgroup analysis stratified by measurement method, high IL-17 level was associated with susceptibility to ICVD in the enzyme-linked immunosorbent assay method subgroup (SMD = 2.24, 95% CI: 0.67–3.80, *P* = 0.005) (Fig. 5).

## Discussion

ICVD is the leading cause of death and disability worldwide, posing a serious threat to human health and with a serious burden on social development. According to a research report in 2017, age-adjusted stroke and ischemic heart disease were the leading causes of years of life lost. Currently, the burden of ICVD is relatively heavy [20–22]. Previous studies have shown that ICVD and its adverse consequences involve inflammatory processes, and many studies have reported a correlation between IL-17, CHD, and stroke. However, previous results regarding the association between IL-17 level and ICVD have been inconsistent. In the present study, we conducted a meta-analysis of published studies to clarify the association between IL-17 level and ICVD. The results showed that among the case–control studies included in this meta-analysis, patients in the case group had higher level of IL-17 compared with healthy controls. This finding suggests that high level of IL-17 may be a risk factor for ICVD. Our research showed a strong association between high level of IL-17 and the development of ICVD. In addition, the subgroup meta-analysis showed that sample size and IL-17 detection methods may be important factors influencing the heterogeneity of previous studies. Further evidence on the involvement of IL-17 in ICVD is needed to clarify the role of IL-17 level as a potential biomarker for ICVD.

IL-17 is secreted by Th17 cells and stimulates various cells to release adhesion molecules and pro-inflammatory cytokines. Studies have found that IL-17 played an important role in various inflammatory diseases by participating in the regulation of chronic inflammation, atherosclerosis, and thrombosis. As the most common type of atherosclerosis, ICVD is linked to IL-17, which can induce a strong inflammatory response and produce molecules important for inflammatory signal transduction, especially cytokines expressed in immune cells. Therefore, upregulation of cytokines can be detected before the occurrence of ICVD [2]. In addition to inducing interferon gamma (IFN-γ), IL-17 can also induce tumor necrosis factor alpha and the expression of IL-6 and its synthesis. These cytokines can promote the inflammatory process, leading to unstable atherosclerotic plaques and thrombosis. From the above analysis, we can conclude that high IL-17 level is associated with the development of ICVD by connecting many types of inflammatory cytokines, leading to changes in atherosclerotic plaque formation and thrombosis and ultimately exacerbating the development of ICVD.

Our study results are consistent with the findings of a study by Jafarzadeh et al, in which patients with ICVD had significantly higher mean serum IL-17 level than healthy controls. In 2009, those authors proposed for the first time that the level of IL-17 in serum can represent the level of intra-coronary artery inflammation and can be used as an independent predictor of ICVD [23]. The pathogenesis may be because vascular endothelial dysfunction, inflammatory response occurs, and immune-related Th17 cells release IL-17, IL-17 can induce apoptosis of vascular endothelial cells, aggravate endothelial dysfunction, and the persistence of inflammation leads to expansion of the degree and scope of coronary artery disease. Local inflammation of the blood vessel wall and various inflammatory mediators reduce the endothelial barrier function, which triggers the multiple cascade release of inflammatory factors, leading to coronary artery calcification, atherosclerotic lesions, and even AMI [24]. Li found that the level of IL-17A around an infarct and cerebrospinal fluid increased significantly after cerebral artery obstruction. This finding may be because IL-17A participates in the inflammatory cascade in various ways, thereby aggravating injury to ischemic brain tissue [25]. Studies on stroke have shown that IL-17 can co-promote cerebral ischemia reperfusion injury through synergistic action with inflammatory mediators. After the acute stage of stroke, owing to vasospasm, endothelial cells are damaged and inflammatory immune cells are activated, which can directly or indirectly lead to an increase in IL-17. It can strengthen the inflammatory response and cause the deposition of subcutaneous immune complex in blood vessels, thereby promoting the formation of thrombosis, aggravating brain tissue damage, and forming a vicious cycle. IL-17A is also involved in the development of atherosclerosis, hypertension, and atrial fibrillation by promoting water and sodium retention and altering myocardial electrophysiology [26]. Other studies have shown that IL-17 can also increase the instability of atherosclerotic plaques and lead to plaque rupture [27].

Considering other related factors that could possibly affect the association between higher IL-17 level and the pathogenesis of ICVD, we conducted a stratified analysis according to race and ethnicity, sample size, and protein measurement method. From the stratified analyses, we concluded that the association was significant in studies from both China and Germany, but there was little such correlation found in a study from the United States, which may be explained by differences in lifestyles among countries. Our results demonstrated that high IL-17 level were strongly linked to ICVD. IL-17 has an important role in promoting the formation of atherosclerotic plaques in intracranial vessels and is a risk factor for ischemic stroke (IS). It is important to clearly understand the pathophysiological effects of IL-17A in different stages of IS and to propose potential therapeutic approaches accordingly. Although there are few interventions targeting IL-17, in view of its special importance, the present study can provide new ideas in the prevention of ICVD. These results suggest that IL-17 is a useful diagnostic and prognostic indicator for ICVD.

This study is the first meta-analysis of available evidence from observational studies investigating the association between IL-17 and ICVD using standardized measures of association to allow for comparison. We pooled data from 6 studies with 701 cases and 255 healthy controls, providing substantial statistical power for the association between IL-17 and ICVD. Based on previous studies, this study is innovative. The data analysis in this study was comprehensive, with data retrieved from four major databases, which allowed for the discovery of statistically significant associations. Nevertheless, the limitations are still important factors to consider when interpreting the results of this study. Our findings were limited by the lack of longitudinal data on IL-17 level from a larger number of ICVD cohorts, as well as clinical findings regarding the specific location of lesions. Therefore, data on the number and location of lesions from larger samples is crucial to more accurately interpret our results. Second, there was a lack of classification of ICVD subtypes; therefore, we could not confirm IL-17 level according to different subtypes. Third, owing to the presence of heterogeneity, the racial and ethnic backgrounds and measurement methods in the included studies may not be comparable.

## Conclusions

In this study, we conducted a meta-analysis to comprehensively assess the correlation between IL-17 level and ICVD. We found that IL-17 level among patients in the case group were higher than those in healthy controls, indicating that high IL-17 level may be a risk factor for ICVD.

### Supplementary Information

**Supplementary Material 1.**

## Data Availability

No datasets were generated or analysed during the current study.

## Abbreviations

ACS: Acute coronary syndrome
SMD: Standardized mean difference
IL-17: Interleukin 17
ICVD: Ischemic cardiovascular disease
SD: Standard deviation
CI: Confidence interval
IV: Inverse variance
SE: Standard error
ELISA: Enzyme-linked immunosorbent assay
FCM: Flow cytometry
df: Degrees of freedom
IFN-γ: Interferon gamma
IS: Ischemic stroke
