# Supplementary material for: Meta-analysis of the association between interleukin-17 and ischemic cardiovascular disease
Source: BMC Cardiovasc Disord. 2024 May 16;24:252. doi: 10.1186/s12872-024-03897-w (PMC11097571; doi:10.1186/s12872-024-03897-w)
Supplement: Supplementary file 1 — Supplementary Material 1. [file 12872_2024_3897_MOESM1_ESM.docx]

| PubMed |
| --- |
| "il 17"[Title/Abstract] AND ((("ischemia"[MeSH Terms] OR "ischemi*"[Title/Abstract]) AND ("Cardiovascular Diseases"[MeSH Terms] OR "cardiovascular disease*"[Title/Abstract])) OR (("acute"[All Fields] OR "acutely"[All Fields] OR "acutes"[All Fields]) AND ("myocardial infarction"[All Fields] OR "myocardial infarction"[MeSH Terms])) OR ("angina, stable"[MeSH Terms] OR "stable angina"[All Fields]) OR ("angina, unstable"[MeSH Terms] OR "unstable angina"[All Fields]) OR ("arrhythmias, cardiac"[MeSH Terms] OR "arrhythmias cardiac"[All Fields] OR "cardiac arrhythmias"[All Fields] OR "arrhythmia*"[All Fields])) |
| Web of Science |
| (("Interleukin 17"[MeSH Terms] OR "IL-17"[Title/Abstract]) AND ((("ischemia"[MeSH Terms] OR "ischemi*"[Title/Abstract]) AND ("Cardiovascular Diseases"[MeSH Terms] OR "cardiovascular disease*"[Title/Abstract])) OR  (("acute"[All Fields] OR "acutely"[All Fields] OR "acutes"[All Fields]) AND ("myocardial infarction"[All Fields] OR "myocardial infarction"[MeSH Terms])) OR ("angina, stable"[MeSH Terms] OR "stable angina"[All Fields]) OR ("angina, unstable"[MeSH Terms] OR "unstable angina"[All Fields]) OR ("arrhythmias, cardiac"[MeSH Terms] OR "arrhythmias cardiac"[All Fields] OR "cardiac arrhythmias"[All Fields] OR "arrhythmia*"[All Fields])) |
| Cochrane Library |
| (("Interleukin 17"[MeSH Terms] OR "Interleukin-17"[Title/Abstract] OR "Interleukin-17 Receptors"[Title/Abstract]) OR "IL-17"[Title/Abstract] OR "IL-17 Receptors"[Title/Abstract] OR "Receptors, Interleukin-17"[Title/Abstract]) AND (("ischemia"[MeSH Terms] OR "ischemi*"[Title/Abstract]) AND ("Cardiovascular Diseases"[MeSH Terms]) OR ("cardiovascular disease*"[Title/Abstract])) OR  (("acute"[All Fields] OR "acutely"[All Fields] OR "acutes"[All Fields]) AND ("myocardial infarction"[All Fields]) OR ("myocardial infarction"[MeSH Terms])) OR ("angina, stable"[MeSH Terms] OR "stable angina"[All Fields]) OR ("angina, unstable"[MeSH Terms] OR "unstable angina"[All Fields]) OR "arrhythmias, cardiac"[MeSH Terms] OR "arrhythmias cardiac"[All Fields] OR ("cardiac arrhythmias"[All Fields] OR ("arrhythmia*"[All Fields])) |
| Embase |
| #1 ((("Interleukin 17"[MeSH Terms]) OR ("IL-17"[Title/Abstract])) OR ("Interleukin-17 Receptors"[Title/Abstract])) OR ("Interleukin-17"[Title/Abstract])  #2 (((("Cardiovascular Diseases"[MeSH Terms]) OR ("cardiovascular disease*"[Title/Abstract])) OR ("acute"[All Fields])) OR ("acutely"[All Fields])) OR ("acutes"[All Fields])  #3 ((((((((("myocardial infarction"[All Fields]) OR ("myocardial infarction"[MeSH Terms])) OR ("angina, stable"[MeSH Terms])) OR ("stable angina"[All Fields])) OR ("angina, unstable"[MeSH Terms])) OR ("unstable angina"[All Fields])) OR ("arrhythmias, cardiac"[MeSH Terms])) OR ("arrhythmias cardiac"[All Fields])) OR ("cardiac arrhythmias"[All Fields])) OR ("arrhythmia*"[All Fields])  #4 #1AND#AND#3 |
